# Pregnancy and birth outcomes of women with intellectual disability in Sweden: a national register study

**DOI:** 10.1111/j.1600-0412.2012.01509.x

**Published:** 2012-12

**Authors:** Berit Höglund, Peter Lindgren, Margareta Larsson

**Affiliations:** Department of Women's and Children's Health, Uppsala UniversityUppsala, Sweden

**Keywords:** Intellectual disability, pregnancy, delivery, midwifery care, national register

## Abstract

*Objective*. To investigate the antenatal health and demographic factors as well as pregnancy and delivery outcomes in women with intellectual disability (ID) in Sweden. *Design.* A population-based register study. *Setting.* The National Patient Register (NPR) linked to the Medical Birth Register (MBR). *Sample.* Women with ID classified as International Classification of Diseases (ICD) 8–10 who gave birth in 1999–2007 (*n* = 326), identified from the NPR linked to the MBR, were compared with all first-time, singleton mothers without ID or any other psychiatric diagnoses during this period in Sweden (*n* = 340 624). *Methods.* Population-based data were extracted from the NPR and the MBR. *Main outcome measures.* Health and socio-demography at first antenatal visit, mode of delivery, pain relief during labor, preterm birth and discharge from hospital. *Results.* A higher proportion of women with ID were teenagers (18.4 vs. 3.3%), obese (20.1 vs. 8.6%) and single (36.6 vs. 6.2%) compared with women without ID, and women with ID smoked more often (27.9 vs. 7.9%). Women with ID had more often a preterm birth (12.2 vs. 6.1%), a cesarean section (CS) (24.5 vs. 17.7%) and used less nitrous oxide as pain relief during labor (59.5 vs. 75.8%). Women with ID had a higher risk for preterm birth [odds ratio (OR) 1.68], CS (OR1.55), non-use of nitrous oxide (OR 1.89) and discharge from hospital to a place other than home (OR 2.24). *Conclusion*. Pregnant women with ID should be considered a risk group suggesting that better tailored pre- and intrapartum care and support are needed for these women.

**Key Message**

Women with intellectual disability have a higher risk of adverse pregnancy outcome and should be considered a risk group. Caregivers need to be aware of this and provide better tailored pre- and intrapartum care and support.

## Introduction

Studies describing pregnancy and childbearing in women with intellectual disability (ID) are few and knowledge is limited. However, Australian studies show that the health of mothers with intellectual limitations is worse than other women's health (1) and one-third of women interviewed reported moderate to severe depression, anxiety, and stress during pregnancy (2). Other studies revealed a higher incidence of preeclampsia (3) and preterm births (28%) (4).

Internationally, according to the World Health Organization, ID is defined as a person with an intelligence quotient below 70, a derogation of adaptive capacity, and a debut before 18 years of age (5). During most of the 20^th^ century, women with ID were institutionalized and/or sterilized to prevent pregnancy. In Sweden, involuntary sterilizations occurred between 1934 and 1975 (6). Currently, there is no legislation prohibiting childbearing in this group; however, negative attitudes towards pregnancies and parenthood exist (7). Few previous Scandinavian studies have investigated pregnancy and childbirth in women with ID. One Swedish study described involuntary sterilizations of women with ID after childbirth or abortion (8).

The aim of the present study was therefore to investigate antenatal health and demographic factors as well as pregnancy and delivery outcomes (mode of delivery, pain relief during labor, preterm birth and discharge from hospital) in women with ID in Sweden compared to women without ID or any psychiatric diagnosis.

## Material and methods

The data were collected from two registers and the use of personal identification numbers allows information between different registers to be linked (9–11). The dataset comprised linkage of the two health registers: the National Patient Register (NPR) and the Medical Birth Register (MBR) (12,13). The NPR has a nationwide coverage of facility care from 1987 and out-patient care was added from 2001. The MBR, established in 1973, contains demographic and medical information from all hospital births and the few home births in Sweden. It covers 98–99% of all births and compiles information related to antenatal and perinatal factors that could be of importance for the health of the infant. The study was approved by the Regional Ethical Committe in Uppsala (327/2007).

All women with an ID diagnosis of International Classification of Diseases (ICD) 8 to 10, chapter V (ICD-8 codes 311–315, ICD-9 codes 317–319, ICD-10 codes F70-F79) were identified from the NPR. This group was linked to MBR, which resulted in a sample including all pregnancies and births to ID women from 1973 to 2006 in Sweden (*n* = 2332 births). To enable a more stringent comparison, only single pregnancies and primiparas were selected for analysis. Data from 1999–2007 were included because, during this period, all pregnancy and delivery variables of interest for the study were represented. To allow comparison, all women without ID or any other psychiatric diagnoses (*n* = 340 624) who had their first child (only singletons) during the same period were identified from the Swedish Medical Birth Register and served as a control group. This resulted in 326 women with ID and 340 624 women without ID or any other psychiatric diagnosis.

For comparison we selected the majority of variables on demographic factors, maternal health, pregnancy and delivery outcomes that were available in MBR during the entire time-period of investigation. Some of these variables had also been used in other studies, which enabled comparisons (1–3,7). We omitted a small number of variables that were not well registered (many missing values) such as smoking in the third trimester. The outcome variables were maternal health and socio-demographic circumstances at registration for antenatal care, mode of delivery, pain relief during labor, preterm birth and discharge from hospital. The proportion of missing values in all variables was similar in the two groups except for ‘working at registration’ where data were missing for 24.5% of women with ID and 14.2% of women without ID. Missing values were excluded from the analysis.

Maternal age was defined as completed years at the time of delivery and cohabiting if the woman lived with the child's father at the time she registered for antenatal care. Body mass index (BMI) ≤ 24.9 was categorized as lean and normal, overweight as BMI 25.0–29.9 and obese as BMI ≥ 30. Gestational age was based on the results of ultrasonography performed early in the second trimester. Preterm birth is defined by the World Health Organization as the delivery of a child before 37 completed weeks of gestation. Discharge to a place other than home means that the woman was not discharged directly to home from hospital but to another care facility. ID was here defined as non-syndromic ID (14).

### Statistical analysis

Statistical analyses were with the SPSS 15.0 software program for Windows (IBM Statistical Package for the Social Sciences). Descriptive statistics were used to describe the frequencies of all the variables presented. The differences between women with ID and women without ID were analyzed by chi-squared test or risk ratios (RR) with 95% confidence intervals (Mantel–Haenszel method). Interval scaled variables were analyzed by comparing means with the independent-samples *t*-test. Variables that differed in the univariate analysis were inserted into a binary logistic regression analysis. To reveal associations between preterm birth, cesarean section (CS), nitrous oxide, discharge to place other than home and ID we used unadjusted binary logistic regression and binary logistic regression adjusted for maternal age, BMI, cohabitation, working, smoking and epilepsy (age was used as a continuous variable).

## Results

At the first antenatal visit more women with ID did not cohabit (36.6%) with the father of the expected child than did women without ID (6.2%) Table 2. More than half of the women with ID did not work (54.1%), compared with 14.3% of the women without ID. In the comparative analysis, the mean age of the women with ID was 24.2 years (range 16–46) and the mean age of the women without ID was 28.3 years (range 11–55) (Table 1). The proportion of teenage births (11–19 years) was higher in women with ID (18.4%) than in women without ID (3.3%). It was more common for women with ID to give birth before the age of 24 years (58.6%) than for women without ID (22.4%). Few women in both groups gave birth at an age of 40 years or older. Most women with ID (88.7%) had attended antenatal care services. The diagnosis preeclampsia was registered in 1.1% of the cases. Diabetes was registered in 0.8% of the cases.

**Table 1:** Characteristics of women without intellectual disability (ID) or any psychiatric diagnosis, and women with ID

	Women without ID, mean *n* = 340 624	Women with ID, mean *n* = 326	*p*-value	Missing data Total %
Age (year)	28.28	24.20	<0.001*	0.3
(range)	(11–55)	(16–46)		
Weight at registration (means in kilogram)	66.67	68.47	0.015*	11.8
(range)	(32–188)	(38–128)		
Body mass index	24.02	25.42	<0.001*	13.6
(range)	(15.01–63.17)	(15.23–45.91)		

* Student's *t*-test.

**Table 2:** Sociodemographic and maternal health data of women without intellectual disability (ID) or any psychiatric diagnosis, and women with ID

	Women without ID (ref)		Women with ID				
					
	*n*	%	*n*	%	Relative risk	95%CI	Missing data Total%
Not cohabiting with the father at registration	19,826	6.2	108	36.6	5.9	4.9–7.1	6.5
Not working at registration	41,708	14.3	133	54.1	3.8	3.2–4.5	14.2
Smoking at registration	24,949	7.9	84	27.9	3.5	2.9–4.4	7.2
Epilepsy	1371	0.4	18	5.5	13.7	8.6–21.8	0.0
Preterm birth (weeks 22–29)	1712	0.5	5	1.5	3.0	1.3–7.3	
Preterm birth (weeks 30–36)	19,055	5.6	35	10.7	1.9	1.4–2.7	
Prolonged gestation (weeks 43–45)	2483	0.7	2	0.6	0.9	0.8–1.1	

CI, confidence interval.

The mean weight at registration for antenatal care was about 2 kg higher in women with ID (68.5 kg) than in women without ID (66.7 kg). The mean BMI at registration was 25.4 and 24.0, respectively. The occurrence of obesity in women with ID was higher (20.1%) than in women without ID (8.6%). The mean weight gain during pregnancy was 13.1 kg (weight at delivery – weight at registration).

More than one-quarter (27.9%) of the women with ID smoked at the first antenatal visit compared with 7.9% of women without ID. Smoking decreased over time in the ID group from 33.8% (1999–2001 and 33.0% (2002–2004) to 19.5% (2005–2007). A medical history of epilepsy was registered 14 times more often for women with ID (5.5%) than for women without ID (0.4%) at the first antenatal visit. For women with ID, 12.2% of pregnancies ended with a preterm birth (<37 weeks of gestation), compared with 6.1% of the women without ID. Over time, preterm births occurred in 23.3% (1999–2001), 19.2% (2002–2004) and 17.3% (2005–2007) of women with ID.

Spontaneous onset of labor occurred in 74.5% of women with ID and 81.5% women without ID (Table 3). Vaginal birth was equally common in both groups. Instrumental delivery by vacuum extraction occurred in 8.9% women with ID and 13.8% in women without ID. CS was more common in women with ID (24.5%) than in women without ID (17.7%). Epidural blockade was administered equally to both groups. However, there was a difference in the use of nitrous oxide: 59.5% in women with ID vs. 75.8% in women without ID. The same difference in the use of nitrous oxide was found in the subgroup of women who had a normal vaginal birth (71.9 vs. 84.1%). The use of nitrous oxide among women with ID increased over time: 51.1% (1999–2001), 56.9% (2002–2004) and 67.7% (2005–2007). The women with ID were more likely to be discharged from the maternity ward directly to a place other than their homes (6.5%) than were women without ID (2.4%) were. The multivariate analysis included statistically significant variables from the univariate analysis (Table 4). An ID diagnosis was associated with all the outcomes investigated.

**Table 3:** Delivery data of women without intellectual disability (ID) or any psychiatric diagnosis, and women with ID

	Women without ID (ref)		Women with ID				
					
	*n*	%	*n*	%	Relative risk	95% CI	Missing data Total%
Cesarean section	60,130	17.7	80	24.5	1.4	1.1–1.7	0.0
Vacuum extraction	47,134	13.8	29	8.9	0.6	0.5–0.9	0.0
Epidural anesthesia	142,664	41.9	123	37.7	1.1	0.9–1.2	0.0
Nitrous oxide	258,265	75.8	194	59.5	0.8	0.7–0.9	0.0
No pharmacological pain relief	17,812	5.2	23	7.1	1.4	0.9–2.0	0.0
No pain relief at all	4644	1.4	4	1.2	0.9	0.3–2.4	0.0
Discharge to place other than home	7995	2.4	21	6.5	2.8	1.8–4.2	1.1

CI, confidence interval.

**Table 4:** Unadjusted odds ratios (95% confidence interval) and adjusted odds ratios (95% confidence interval) for preterm birth, cesarean section, non-use of nitrous oxide and discharge to place other than home in women with ID vs. women without ID

	Women with ID vs. women without ID	
	Crude OR (95%CI)	Adjusted OR (95%CI)
Preterm birth	2.15 (1.55–3.00)	1.68 (1.06–2.67)
Cesarean section	1.52 (1.18–1.95)	1.55 (1.11–2.17)
Non-use of nitrous oxide	2.13 (1.71–2.66)	1.89 (1.43–2.50)
Discharge to other place than home	2.88 (1.85–4.49)	2.24 (1.22–4.17)

Adjusted for maternal age, obesity, cohabitation, working, smoking and epilepsy. CI, confidence interval.

## Discussion

This study shows several differences between women with ID and women without ID during pregnancy and childbirth, e.g. a higher proportion of teenagers, obesity and single status among women with ID. Women with ID more often had preterm births and CS. Women with ID used less nitrous oxide as pain relief during delivery than women without ID did, and were more often discharged from the maternity ward to a place other than home. Moreover, there was an overrepresentation of smokers among women with ID at the first antenatal visit.

This study was prospective, based on a standardized collection of data gathered at maternal care units and hospitals; therefore, recall bias was avoided. Maternal care is free of charge and home deliveries are rare in Sweden, thus, selection bias is unlikely. A weakness with the Swedish MBR is the lack of systematic documentation of some variables (11). Sources of errors include incorrect documentation in medical records, changes in medical records, missing records, and deficient transmission into the Swedish MBR. Medical records on pregnancy and childbirth do not include education level and housing condition variables and these data are therefore lacking in MBR. If these variables could be included in medical records, the MBR data would be strengthened. Some characteristics and socio-demographic variables had more missing values than others had, but this was balanced by the large amount of data.

The NPR includes women with the diagnosis ID. The ID diagnosis includes the classifications: mild, moderate, severe, profound, other and non-specific ID. In this study, information about the etiological factor and exclusion of the psychotic group or the severity in women with ID were not known. In the group of women without ID, all psychiatric diagnoses were excluded to eliminate incorrect adjacent classifications; this could mean this group was healthier than the general population. The results in this study should be compared with similar studies and may not be generalizable to all populations. However, in populations with a similar socio-demographic structure, such as in Sweden, similar results would be expected.

More women with ID (36.6%) did not cohabit with the father at the first antenatal visit compared with women without ID (6.2%). One reason could be more pregnancies in women with ID were unplanned or the pregnancies occurred before cohabitation. Women with ID (54.1%) worked less than women without ID (14.3%). Even so, there is a possibility this proportion was overestimated, as data for one-quarter of the women were missing, possibly indicating lack of work in these women. Unpaid work and daily occupational training based on governmental funding can be registered as ‘work’. Generally, in Sweden, people with ID are less often gainfully employed (27.6%) than the general population (75.1%) (15,16). A medical history of epilepsy was more common in women with ID (5.5%) than in women without ID (0.4%), and a higher prevalence of epilepsy is more associated with ID than in the general population (17,18).

During pregnancy, women with ID were on average four years younger than women without ID were. One reason could be that women with ID did not have the level of education or professional work that often competes with childbearing. Alternatively, they may have wished to become a mother early in life. However, there could be other reasons for not using contraceptives, e.g. low compliance with contraceptive methods, side effects, inconvenience, no steady partner for the moment, and poor economy. In the UK, women with ID have limited knowledge about sexual health issues and approximately half of the women lack basic knowledge about reproduction (19): the prevalence of non-contraceptive use is high (40.8%) (20).

More women with ID were obese than women without ID. An explanation may be that women with ID registered later in the pregnancy with antenatal care than women without ID, and had already gained some weight due to the pregnancy. However, the average weight gain between registration with antenatal services and delivery (13.1 kg) indicated a substantial period must have elapsed between the two registered weights. Obesity is a key risk factor for ill-health among women with ID (21,22).

The overrepresentation of smokers among women with ID could be explained partly by their younger age at childbirth, as younger women in Sweden smoke more than older women do (23). However, it is also possible the women with ID did not receive ‘tailored’ information about smoking, did not understand the smoking information, did not want to stop smoking during pregnancy, or did not receive adequate support to be able to avoid smoking. In Finland, the prevalence of smoking in early pregnancy (25.7%) is higher among young, primiparas, unmarried women with preterm birth (24). As tobacco is the only substance reported in the MBR, the use of alcohol or other drugs among these women could not be assessed. However, it is assumed women with ID live in more deprived settings where alcohol and substance abuse are more prevalent (25).

A higher proportion of women with ID (12.2%) had a gestation length of less than 37 full weeks, compared with 6.1% in the women without ID, and the proportion of preterm deliveries was constant over time. In Australia, the proportion of preterm births among women with ID is 28% (4), although the causes of preterm births are unknown. Reasons for shorter gestation length could be that women with ID have more difficulty in understanding and interpreting signs and symptoms of pregnancy complications such as premature contractions. Other medical reasons include preeclampsia, which is more prevalent during pregnancy among women with ID than among women without ID (OR = 2.85) (3). However, in our data retrieved from MBR a diagnosis of preeclampsia was only reported in 1.1% of women with ID, possibly due to underreporting. A shorter gestational length may pose different problems for women with ID. If labor starts unexpectedly, they may not have received sufficient or appropriate information and are thus unprepared, which can cause more anxiety and stress during delivery, and lead to more CSs being performed on medical as well as humanitarian indications.

During labor, nitrous oxide is an effective and safe analgesic and is the most common pain relief in Sweden (26,27). Totally, women with ID used this method (59.5%) less than women without ID did (75.8%) and the same difference was found in the subgroup of women who had a normal vaginal birth (71.9 vs. 84.1%). This could reflect less need for pain relief or the lack of ability to express the need. Alternatively, the midwife might not have interpreted the women's difficulty in expressing their need and misjudged or underestimated the women's need for pain relief. Another possible explanation is that these women had less knowledge about pain relief during delivery and consequently did not express any wishes. Epidural anesthesia is the most potent analgesia during delivery and the use of epidural analgesia did not differ between the two groups. Severe pain requiring epidural analgesia may be more obvious and easier for women to express and for midwives to understand and handle.

Spontaneous onset of labor was more common in women without ID (81.5%) than in women with ID (74.5%); however, delivery ended more often with a CS in women with ID (24.5%) than in women without ID (17.7%). The indications could be either medical or psychosocial for both elective and acute CS. Severe preeclampsia in primiparas is associated with the child being small for gestational age (28) and this condition may lead to CS. Further, moderate to severe depression, substance abuse, anxiety and stress during pregnancy (2), and specific types of anxiety, e.g. psychosocial stress, family functioning and fear of childbirth, may have associations with CS (29). Another reason for preterm by itself can include presentations other than vertex presentations that exclude vaginal delivery. Vacuum extraction demands more participation from the woman, which may be difficult to obtain from women with ID, or professionals may consider this collaboration is difficult to establish.

Women with ID were more often discharged from the maternity ward to place other than home than were women without ID. Children born to women with ID were more frequently admitted to a neonatal intensive care unit (OR = 2.51) (3) and, as gestation length was shorter in women with ID, the children might have needed more neonatal care. Another reason could be that women with ID needed medical care or referral to an institution for planning and assessment of bonding with their babies and training in parenting skills.

Women with ID are a neglected group in society, yet they still have to elucidate their feelings and needs, as every woman does. They want to be treated with respect and consideration, to have the opportunity to express their wishes and to control their lives from their own point of view. The focus has been on others’ reactions to the pregnancy rather than on the pregnant women themselves (7). To understand the needs and experiences of women with ID in childbearing, it is necessary to recognize them as women instead of medical problems and to acknowledge their human rights (30): they have a right to be heard. This study confirmed women with ID are vulnerable during pregnancy and delivery. Further studies should investigate if their newborn children have a similar increased vulnerability.

## Conclusion

Women with ID had more preterm births, used less nitrous oxide as pain relief in labor, and the delivery ended more often with CS. A higher proportion of women with ID smoked at the first antenatal visit. These findings imply that women with ID should be regarded as a risk group. We suggest the knowledge and skills of prepartum and intrapartum caregivers should be improved to enhance more accessible, interactive and better-tailored information, support and care for women with ID.

## Abbreviations

BMI: body mass index
CS: cesarean section
ICD: International Classification of Diseases
ID: intellectual disability
MBR: Medical Birth Register
NPR: National Patient Register
OR: odds ratio
RR: relative risk
